# Pedestrian Detection Using Multispectral Images and a Deep Neural Network

**DOI:** 10.3390/s21072536

**Published:** 2021-04-04

**Authors:** Jason Nataprawira, Yanlei Gu, Igor Goncharenko, Shunsuke Kamijo

**Affiliations:** 1 College of Information Science and Engineering, Ritsumeikan University, Shiga 525-8577, Japan; jason.nataprawira@kanisius.org (J.N.); igor@fc.ritsumei.ac.jp (I.G.); 2 Institute of Industrial Science, The University of Tokyo, Tokyo 153-8505, Japan; kamijo@iis.u-tokyo.ac.jp

**Keywords:** pedestrian detection, different lighting conditions, deep neural network, multispectral images, processing time

## Abstract

Pedestrian fatalities and injuries most likely occur in vehicle-pedestrian crashes. Meanwhile, engineers have tried to reduce the problems by developing a pedestrian detection function in Advanced Driver-Assistance Systems (ADAS) and autonomous vehicles. However, the system is still not perfect. A remaining problem in pedestrian detection is the performance reduction at nighttime, although pedestrian detection should work well regardless of lighting conditions. This study presents an evaluation of pedestrian detection performance in different lighting conditions, then proposes to adopt multispectral image and deep neural network to improve the detection accuracy. In the evaluation, different image sources including RGB, thermal, and multispectral format are compared for the performance of the pedestrian detection. In addition, the optimizations of the architecture of the deep neural network are performed to achieve high accuracy and short processing time in the pedestrian detection task. The result implies that using multispectral images is the best solution for pedestrian detection at different lighting conditions. The proposed deep neural network accomplishes a 6.9% improvement in pedestrian detection accuracy compared to the baseline method. Moreover, the optimization for processing time indicates that it is possible to reduce 22.76% processing time by only sacrificing 2% detection accuracy.

## 1. Introduction

### 1.1. Pedestrian Safety and Challenges in Pedestrian Detection

Pedestrian fatalities and injuries are significant issues in modern traffic systems. In the United States 16% of the total traffic fatalities recorded in 2017 were pedestrians [1]. In the same year, pedestrians also accounted for 17% of the total traffic fatalities in the United Kingdom [1]. Those numbers do not include the data from the developing countries, which presumably can be higher than those reported by developed countries. Hence, pedestrian safety is still an important unsolved issue.

Many engineers have tried to solve these issues through new inventions. A system called Advanced Driving Assistance System (ADAS) has been developed for car users to prevent unexpected accidents from happening. This system is equipped with many features to support the safety of passengers, drivers, and the surroundings. One installed feature is called pedestrian detection. Engineers also include this feature to autonomous vehicles. However, the system is still not perfect. In 2018, a fatality occurred in an autonomous vehicle accident [2]. This accident left engineers with the big task of ensuring the system would work properly, so such an accident will not happen again in the future. This research is conducted to enhance pedestrian safety through the pedestrian detection domain.

Human eyes cannot adapt very well to dark environments. This is because human eyes are sensitive to light sources. It is also believed that one of the major causes of nighttime accidents is the incapability of human eyes to perceive things in the dark. 75% of traffic incidents involving pedestrians happened during dark conditions in 2017 [3]. Aside from that fact, lighting conditions have become an obstacle in the pedestrian detection field [4]. An automatic braking emergency experiment was run by the American Automobile Association (AAA). The results indicated that pedestrian detection was ineffective during nighttime conditions [5]. Four scenarios were examined with no ambient light conditions and when only a low-beam car light was used for lightening the environment. In those experiments, four different cars did not decelerate correctly in any of the four tests. As a result, this indicates that pedestrian detection does not behave as expected under nighttime conditions, although conceptually it should perform well under any lighting conditions. 

### 1.2. Sensors for Pedestrian Detection 

Pedestrian detection systems can be developed based on different sensor solutions, such as Light Detection and Ranging (LiDAR) sensors, Millimeter-Wave Radar (MWR) sensors, cameras, and fusion of data from different sensors. In a LiDAR-based pedestrian detection system, a dense point cloud data can be observed for a close pedestrian, however, it is difficult to obtain the detailed shape information for distant pedestrians because the obtained point clouds are sparse [6]. A radar-based sensor solution for pedestrian detection also has some drawbacks to overcome. For instance, a pedestrian has a much lower radar cross-section than a passenger car. Therefore, it is harder to identify the objects in the received signal spectrum. A correct distinction becomes especially difficult due to resolution capabilities when pedestrians are in the proximity of cars [7]. In contrast, denser observation information can be obtained by a camera compared to LiDAR and MWR sensors and it is possible to recognize distant (over 100 m away) objects by selecting an appropriate lens [6]. The rich texture information provided by camera sensors can be used not only for pedestrian detection, but also for analyzing the posture and intention of pedestrians in the advanced pedestrian safety function. 

Aside from the standalone sensor solutions, fusion of the different sensors is also proposed for pedestrian detection. The integration of different sensors can bring redundancy and complementary characteristics of sensors for improving the detection system’s reliability and accuracy [8]. However, the camera is the most commonly used sensor as the basis for a pedestrian detection system [9], and camera-based pedestrian detection systems cannot avoid the problem of detection in nighttime conditions. Therefore, this study focuses on camera-based pedestrian detection systems.

### 1.3. Computer Vision for Pedestrian Detection

In the computer vision field, the development of pedestrian detection algorithms had a long history well before the beginning of deep neural network (DNN) usage for pedestrian detection. The research was pioneered in 2003 when improved Haar-like features [10] were applied to object detection. The researchers combined information from Haar-like features with motion information. Then, Histograms of Oriented Gradients (HOGs) were created by [11] for the pedestrian detection task. This technique classifies objects by getting feature information from the image through edge direction distributions [12]. Furthermore, a support vector machine (SVM) was applied as the classifier in [11]. Additionally, Zhang et al. [13] developed a new feature set called “Shapelet” with the AdaBoost classifier to detect pedestrians.

Until the current time, DNNs have been developed for many applications, such as civil engineering [14], electrical engineering [15], petroleum engineering [16], industrial engineering [17], and computer vision. A DNN for object detection is mostly known as a convolutional neural network (CNN). In general, there are two categories: two-stage detector or sparse prediction type, and one-stage detector or dense prediction. A two-stage detector has separate networks for selecting region information, classification and localization. In contrast, a one-stage detector unites all tasks into a single network architecture.

The two-stage detector was first invented by Girshick et al. [18]. The first development still used a conventional way, for example by generating fixed 2000 region proposals over an image. Afterward, localization and classification were executed on the proposals. Furthermore, it was improved by the release of Fast Region-Convolutional Neural Network (Fast R-CNN) [19] and Faster R-CNN [20]. Fast R-CNN improves R-CNN by letting the whole image be used to find region proposals. Also, the classification is done through two fully connected layers: softmax and bbox regressor. Moreover, Faster R-CNN introduces the Region Proposal Network (RPN) on top of Fast R-CNN architecture. RPN is needed for obtaining the most possible region proposals. Another significant improvement is the anchor box usage. The anchor box becomes the base of the one-stage detector, as it allows the model to detect objects faster and more accurately. 

Two famous one-stage detector algorithms are Single Shot Detector (SSD) [21] and You Look Only Once (YOLO) [22,23,24]. The idea of a one-stage detector is to remove the region proposal layer, so the feature extraction and classification can be performed in a single network architecture. The initial prediction is made by using anchor boxes. A lot of bounding boxes can be drawn over an image. Therefore, to make the best prediction, Non-Max Suppression (NMS) is used. The confidence score is also computed on each bounding box. This score is measured to represent the object’s existence on that particular bounding box. At the last step, objects will be classified through a 1 × 1 convolutional layer. One noticeable difference between YOLO and SSD is the initial anchor numbers. YOLO implements k-mean clustering to generate initial anchor numbers, whereas SSD fixes their numbers. The R-CNN family networks, YOLO and SSD have been widely used for pedestrian detection during the daytime [25,26,27].

### 1.4. Pedestrian Detection at Different Lighting Conditions

Despite the development of advanced detection algorithms, one problem remains unsolved in pedestrian detection domain. Lighting conditions are considered one of the major problems [4]. As mentioned above, visibility is impaired at nighttime, because of the limited lighting sources, especially on streets. Several approaches were proposed to enhance vision at nighttime. 

The first approach is the use of infrared sensors. Two types used in the pedestrian detection applications are Far Infrared (FIR) and Near Infrared (NIR). Piniarski et al. [28] utilized FIR and NIR for recognizing pedestrians under low-light conditions. They implemented Connected Component Labeling (CCL) for the feature proposals, HOG for the feature extraction, and SVM for the classifier. A Forward-Looking Infrared (FLIR) camera was employed by Sun et al. [29] along with the use of Haar-like features and AdaBoost classifiers. Some researchers also implemented NIR [30,31,32], even though it was claimed the longer wavelength will make the detection more robust to detect pedestrians [4].

The second approach was proposed by Chebrolu and Kumar [33]. They developed a brightness awareness model. This model can recognize the image ambiance to switch between a daytime model and a nighttime model. For the daytime environment, the model uses an RGB-based model, whereas for the nighttime environment, the model uses a thermal-based model.

The last approach is called the fusion or multispectral method. It started to be popular when the multispectral pedestrian dataset was released [4]. Several DNN algorithms have been tested for applying fusion datasets, such as SSD [34], RPN combined with Boosted Decision Trees [35], and the combination of CNN and Support Vector Networks (SVR) [36]. 

### 1.5. Contributions on Pedestrian Detection

Even though different solutions have been proposed for pedestrian detection, which image source is the best choice for pedestrian detection in different lighting conditions re-mains unanswered. The novelty of this paper is to compare the performance of pedestrian detection with different image sources, and further improve the pedestrian detection performance from both accuracy and processing time aspects. 

**Figure 1:**
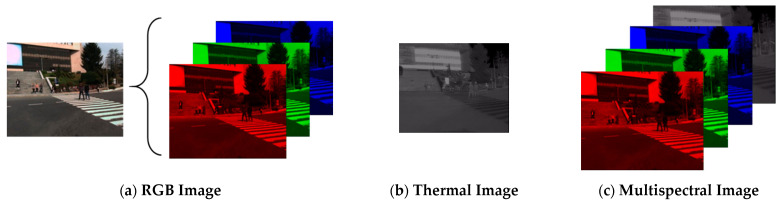
Three image sources are utilized: (**a**) RGB, (**b**) thermal and (**c**) multispectral images. The RGB image and the thermal image are taken by the dataset. The RGB image can be decomposed into red, green and blue channels. Thermal image only has one channel. Multispectral image consists of a concatenation of three channels of RGB image and one channel of thermal image.

In this research, an evaluation and comparison of pedestrian detection on different image sources is proposed as the first contribution. It covers three types of image sources: RGB image, thermal image, and multispectral image. As shown in Figure 1, a multispectral image is an aggregate image of RGB and thermal images. The RGB image consists of three major color channels (red, green, and blue), whereas the thermal image generally consists of one channel. As a result, a multispectral image has four channels.

Another contribution of this research is to optimize the DNN algorithm called YOLO v3 [24]. Firstly, an optimization is applied to improve the accuracy of YOLO v3 when doing pedestrian detection tasks. Generally, the YOLO v3 architecture has been created to handle objects in three resolutions. However, in the real implementation, pedestrian sizes can be diverse assuming the pedestrian is captured by a camera. If a pedestrian stands too far from the camera, he or she will look small in the image. On the contrary, if a pedestrian stands close enough to the camera, he or she will look big in the image. Therefore, the YOLO v3 architecture is modified to improve the detection performance for smaller pedestrians.

Despite improving the accuracy, an optimization on processing time while maintaining the performance is also conducted as the third contribution. Most DNN algorithms still require expensive computational hardware to run the algorithm. Because this system will be implemented in the autonomous vehicle, the algorithm needs to work efficiently, but still maintain a fair performance. Several compressed models are discussed in this research. The best model will be concluded to recommend a balanced model for small-scale applications. 

The initial results of this research have been published in an international conference [37]. Compared to the conference paper, optimizations of the architecture of deep neural network are newly proposed. In addition, more details of experimental results are presented in this paper. This paper is a summary of the undergraduate graduation research of the first author [38]. 

## 2. Pedestrian Detection in Different Lighting Conditions

### 2.1. Multispectral Image Use for YOLO

To understand which is the best solution for pedestrian detection under different lighting conditions, this study compares the performance when using different image sources for pedestrian detection. In addition to RGB and thermal images, this research also utilizes multispectral images for pedestrian detection in different lighting conditions. The system is designed by merging RGB image and thermal image as depicted in Figure 1. RGB color channels take the front three channels, while the grayscale channel from a thermal channel is placed behind the RGB color channels. Thus, it produces a 4-channel multispectral image. It is possible to pre-process the RGB and thermal images with fuzzy or neuro-fuzzy techniques in order to improve the image quality before applying the proposed methodology [39]. This research focuses on the comparison of the performance of pedestrian detection using different image sources. To be fair, all types of images are input to the developed algorithm without pre-processing.

**Figure 2:**
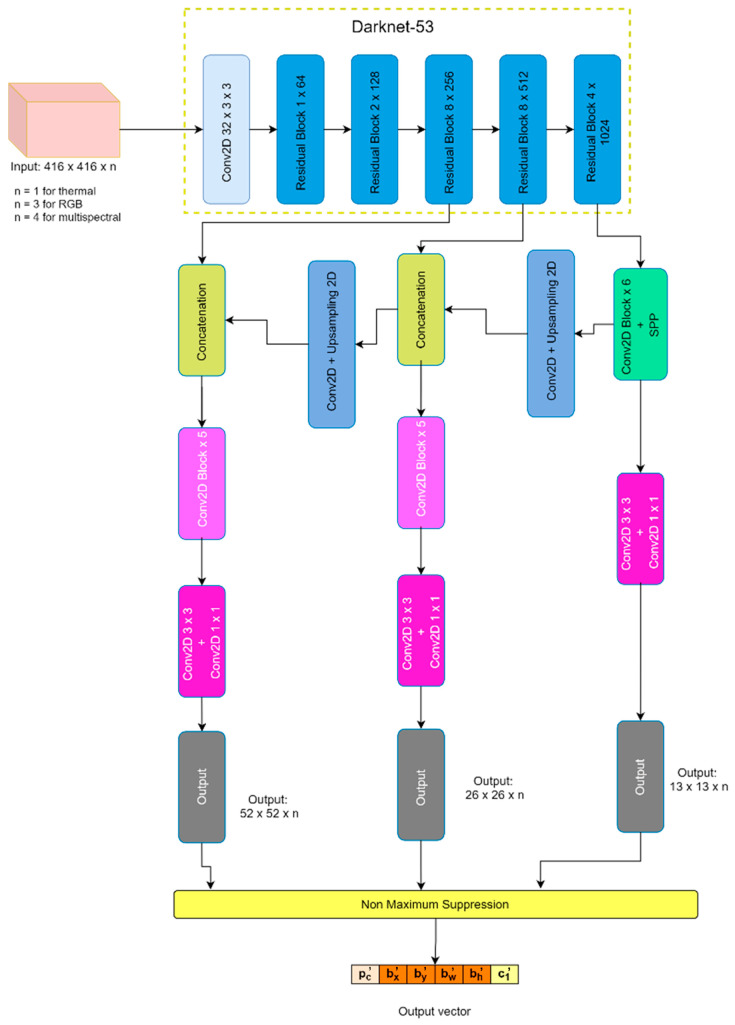
The system design of YOLO v3 with various image sources. The default input image size is 416 × 416 × n. n denotes the number of channels of the input image. Depending on the image sources, 1-channel is used for the thermal image, 3-channel is used for the RGB image, and 4-channel is used for the multispectral image. For all channels, all images are passed into the same architecture after the input. In the output vector, $b_{x}^{\prime }$ and $b_{y}^{\prime }$ indicate the centroid of the detected object, $b_{w}^{\prime }$ and $b_{h}^{\prime }$ are the width and height of detected object, $p_{c}^{\prime }\;$ and $c_{1}^{\prime }$ denote the probability of object existence and what class this detected object corresponds to.

Various methods can be suggested for producing multispectral images. In this case, the images are merged at the input stage. RGB and thermal images are loaded simultaneously, then they are merged to produce the new 4-channel image. Eventually, the newly generated image will be fetched into the YOLO v3. Similarly, to compare the performance of multispectral images, RGB and thermal images are also used for training independently with the same method. For RGB image, 3-channel is used, and for thermal image 1-channel is used. Therefore, there are no differences regarding the DNN algorithm for different image sources. The system design can be seen in Figure 2. 

YOLO is known to be one of the best one-stage detector algorithms. This paper adopts YOLO as the basic framework to evaluate detection performance and develop a deep neural network. The experiment is conducted using the YOLO v3 open source code written in the PyTorch library [40]. This source code is modified to allow YOLO v3 loading images depending on the color channels. 416 × 416 image size is set to prevent any mislearning while training the model. Each image source-based model is trained and validated independently. The mathematics behind the YOLO algorithm are explained in the next subsection. Also, the accuracy will be compared, which is explained in the Experimental section.

### 2.2. Optimization of Deep Neural Network for Improving Detection Accuracy

YOLO detects an object by using the feature called anchor box. This anchor box is a template bounding box with different pre-defined aspect ratios. These aspect ratios are generated before training by running K-mean clustering on the whole training dataset. The predicted bounding box of the detected object is represented by the anchor box. YOLO analyzes an object within an image by dividing the image into grids. Each grid has three anchor boxes, where each anchor box owns the same center point. Once those anchors are defined, it is possible to calculate the Intersection over Union (IoU) between the given ground truth box and each anchor box. One of the purposes of training is to optimize the parameters in YOLO model for minimizing the difference between the predicted bounding box and ground truth box. The first two components in loss function of YOLO are related to this purpose.

The first loss component is for bounding box centroid where it is written as:

(1) $$xy\_loss\;=\;\lambda_{coord}*\sum MSE\left(\left(t_{x},t_{y}\right),\;\left(t_{x}^{\prime },t_{y}^{\prime }\right)\right)*obj\_mask$$

where *λ_coord_* represents the weight for the centroid loss component, MSE is the Mean Squared Error. $\left(t_{x},t_{y}\right)$ is the relative centroid location from the ground truth, $\left(t_{x}^{\prime },t_{y}^{\prime }\right)$ is the predicted centroid of object from YOLO model. obj_mask indicates if there is an object or not, the value of obj_mask is either 1 or 0. The smaller this loss is, the closer the centroids of prediction to the ground truth are. $\left(t_{x},t_{y}\right)$ is not the absolute centroid location from the ground truth, the values of these two parameters need to be calculated from the following two equations:

(2) $$b_{x}\;=\;\sigma \left(t_{x}\right)+c_{x}$$

(3) $$b_{y}\;=\;\sigma \left(t_{y}\right)+c_{y}$$

where $b_{x}$ and $b_{y}$ are the absolute coordinates of object, *σ* is the sigmoid function, and $c_{x}$ and $c_{y}$ represent absolute location of the top-left corner of the current grid cell. In the output vector of YOLO model, the $b_{x}^{\prime }$ and $b_{y}^{\prime }\;$can be calculated by taking $t_{x}^{\prime }$ and $t_{y}^{\prime }$ into Equations (2) and (3).

Furthermore, the second loss components are the width and height loss, written as:

(4) $$wh\_loss\;=\;\lambda_{coord}*\sum MSE\left(\left(t_{w},t_{h}\right),\;\left(t_{w}^{\prime },t_{h}^{\prime }\right)\right)*obj\_mask$$

where the definitions of the parameters are similar to that in Equation (1). $\left(t_{w},t_{h}\right)$ represents the relative scale offsets of the ground truth box compared with a particular anchor box. $\left(t_{w}^{\prime },t_{h}^{\prime }\right)$ is the relative scale offsets of the predicted box compared with a particular anchor box. The parameters inside MSE are calculated differently as described below:

(5) $$b_{w}=p_{w}*e^{t_{w}}$$

(6) $$b_{h}=p_{h}*e^{t_{h}}$$

where $b_{w}$ and $b_{h}$ are the absolute width and height of the bounding box of the entire image, $p_{w}$ and $p_{h}$ are the absolute value of the width and the height of the anchor box. Exp is taken because $t_{w}$ and $t_{h}$ can result in negative number, so exp function inverts the number into positive as width and height cannot be negative. The smaller this loss is, the closer the scale of prediction and ground truth are. In the output vector of YOLO model, the $b_{w}^{\prime }$ and $b_{h}^{\prime }\;$can be calculated by taking $t_{w}^{\prime }$ and $t_{h}^{\prime }$ into Equations (5) and (6).

In addition to the accuracy of the predicted bounding box, the objectness and non-objectness scores are also considered in the definition of loss function. The “objectness” denotes how likely is there an object in the current cell. The Equations (7) and (8) are the objectness and non-objectness loss.

(7) $$object\_loss=\sum BCE\left(p_{c},\;p_{c}^{\prime }\right)*obj\_mask$$

(8) $$no\_obj\_loss\;=\;\lambda_{no\_obj}\sum BCE\left(p_{c},\;p_{c}^{\prime }\right)*\left(1-obj\_mask\right)*ignore\_mask$$

where object_loss stands for the object loss and no_obj_loss stands for the non-existence object loss. *p_c_* value is the objectness value when the object exists in ground truth, for example it returns 1 when the object exists and otherwise 0. $p_{c}^{\prime }$ is the predicted probability of object existence. By considering the object loss, the network can learn to detect a region of interest. Non-existent object loss is also necessary to avoid the network keeps providing many false positive detections. $\lambda_{no\_obj}$ is the weight for the non-objectness loss component. ignore_mask can insure false positive detections are penalized when the overlap between predicted box and the ground truth box is not too large. Here, BCE means Binary Cross Entropy. 

The classification loss is the final component in the loss function. The classification loss also uses the BCE as described below:

(9) $$class\_loss=\sum BCE\left(c_{1},\;c_{1}^{\prime }\right)*obj\_mask$$

where $c_{1}$ corresponds to the class label in the ground truth, and $c_{1}^{\prime }$ is the predicted class label. The loss function of the employed YOLO model is a summation of Equations (1), (4), (7)–(9).

Referring to Figure 2, YOLO v3 has three feature extraction which leads to three different outputs. For an image of 416 × 416 size, YOLO v3 will produce 13 × 13, 26 × 26, and 52 × 52 outputs. YOLO analyzes an object within an image by dividing the image into grids. Therefore, as the grid size becomes larger, the particular image pixel can give feature information of the smaller objects to the model. 13 × 13 feature output detects the large objects, 26 × 26 feature output detects the mid-sized objects, and 52 × 52 feature output detects the small objects. 

**Figure 3:**
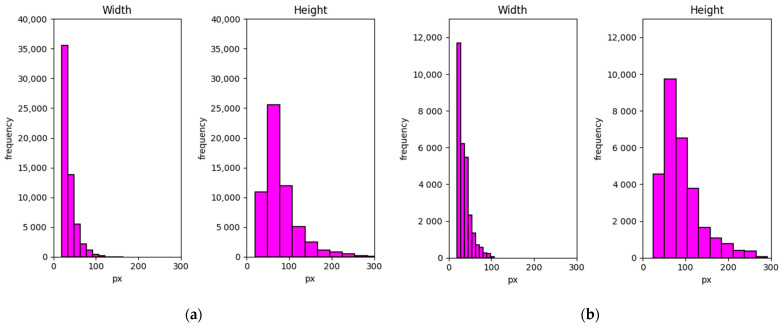
Pedestrian scale distribution in (**a**) daytime and (**b**) nighttime.

Generally, YOLO can recognize objects in a certain range of scales quite well. For the pedestrian detection task, the case can be slightly more complicated. Figure 3 shows the pedestrian scale distribution in the dataset used for this research [4]. In the real situation, the pedestrian sizes vary in both width and height. Assuming this system will be implemented in the car with a camera, a pedestrian can look very small if he or she stands far from the car. On the other hand, if a pedestrian stands close enough to the car, he or she will look large in the image. The ratio between the large pedestrian and small pedestrian is about 20 times. Such large-scale differences should be considered when YOLO is employed for pedestrian detection task. Also, the pedestrian scale distribution indicates that the small pedestrian appears with high frequency. Therefore, this research proposes to optimize the architecture of YOLO with considering the detection of small pedestrians. 

**Figure 4:**
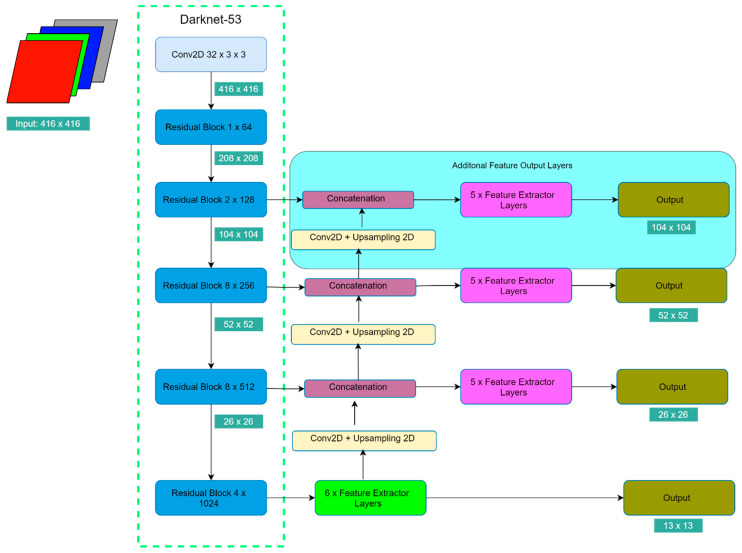
The optimized YOLO v3 architecture for improving detection accuracy. YOLO works by dividing the image into S × S grids. The output of the original YOLO has three resolution of grids: 13 × 13, 26 × 26 and 52 × 52, whereas it does not cover the small pedestrian well. Therefore, a new additional feature output is proposed, so it can handle smaller pedestrians with the grid size of 104 × 104. This deep neural network is named as YOLO-4L.

To accomplish this proposal, additional feature output layers are added. In the original YOLO v3, feature output layers are attached in the 3rd, 4th, and 5th residual blocks. Each feature output layer is responsible for small objects until large objects in ascending order. As a result, to enhance the capability of detecting smaller objects, feature output layers are added from the 2nd residual. The additional feature output layer is the same as the feature output layer in the 3rd and the 4th residual block. The 5th residual block’s feature layer contains a Spatial Pyramid Pooling (SPP) structure. As an illustration, Figure 4 shows the architecture of the optimized YOLO v3. The default image size is 416 × 416. The green cell indicates the image size reduction of the 416 × 416 size. The deep neural network in Figure 4 is named as YOLO-4L.

Additionally, a unified model is developed, so one model can work for both daytime and nighttime conditions. The unified model development is beneficial. It is because the model does not have to switch between the daytime model or the nighttime model. In the real implementation, this switching time can take a longer processing time when detecting pedestrians. Furthermore, a unified model is lighter than having 2 models for different conditions, since there is only one model needed for both daytime and nighttime scenarios.

### 2.3. Optimization Deep Neural Network for Reducing Processing Time

Researchers in the object detection field always aim to achieve the highest accuracy. However, another aspect is also important, that is processing time. The model can be considered good enough when it can reach a higher accuracy than its predecessors or similar model for the same task. Nevertheless, in the pedestrian detection domain, it is also important for the model to run in a small-scale device, especially a car. A car does not have infinite power resources like a supercomputer. Therefore, this study tries to improve processing time from the previous accuracy optimization without sacrificing too much accuracy.

Many methods can be applied for increasing the processing time. Compressing the network architecture is the most common method [41,42]. Based on that, four compressed models are suggested and tested. From the four models, the modification can be classified into two steps. The first step is reducing the feature output layers. Referring to Figure 4, there are five feature extractor layers and six feature extractor layers for the last residual block. This feature extractor length is made originally to detect up to 80 different kinds of objects. In this scenario, there is only a pedestrian who needs to be recognized. Consequently, the assumption is come up that for a single type object, deep feature extractor layers are unnecessary. As a replacement, each feature extractor layer only has one pair of a 3 × 3 convolutional layer and 1 × 1 convolutional layer. For the feature extractor layer that contains an SPP module, two pairs of 3 × 3 convolutional layer and 1 × 1 convolutional layer are removed. As a summary, the first compressed model (YOLO-4L-C1) implements the aforementioned modifications.

The second modification step (YOLO-4L-C2) is reducing the residual block structures. There are so many repeated convolutional layers, especially in the 3rd and the 4th residual block. The same reason goes for these repeated convolutional layers. Therefore, the second compressed model is reducing half number of the 3rd and the 4th residual block, but still, keeping the feature output layers as the original layout. From 5 residual blocks, the 3rd and the 4th residual blocks are chosen, because they have the deepest residual block of all residual blocks. Furthermore, the third compressed model (YOLO-4L-C3) combines the first and the second compressed models. Finally, the fourth compressed (YOLO-4L-C4) model is reducing half of the 4th and the 5th residual block from the third compressed model. The reason is that the mid-size and the large pedestrians can be handled quite well in the previous setup. Therefore, to seek the influence of these two residual blocks, the fourth compressed model is designed. All comparisons can be referred to Table 1 below. The modification parts are marked by the bold fonts.

## 3. Experimental Results

### 3.1. Dataset and Experiment Setup

This research requires a specific dataset with synchronized RGB and thermal images. Although until now there have been various pedestrian datasets out there, there are a few datasets that are suitable for this particular task. The KAIST Multispectral Pedestrian Dataset [4] is chosen in this research.

The advantage of this dataset is that the dataset was all recorded in a multispectral format. To generate a multispectral-image format, the researchers developed a device with supplementary components. An RGB camera called PointGrey Flea 3 was used. Similarly, a FLIR A-35 thermal camera was attached, aligned to the PointGrey Flea 3. The RGB camera is able to generate 640 × 480 pixels resolution with a 103.6° vertical field of view. In contrast, the thermal camera has 320 × 256 pixels resolution with a 39° vertical field of view. Moreover, several additional tools were required, for example a beam splitter and a three-axis jig. A beam splitter was utilized to obtain reflected lights from the transmitted thermal band of the incident light. Consequently, the image datasets are generated parallel to each other.

In total there are more than 50,000 pedestrians in the dataset split into training and testing datasets as shown in Figure 3. Three scenarios were recorded: campus, road, and downtown. Each scenario has three different labels: person, people, and cyclist based on the Caltech dataset format [43]. However, because these three labels data are unbalanced, so only the person label is used in this research.

In all experiments, an Intel Xeon E5-1650 v4 3.60 GHz clock speed 6 cores 12 threads acted as the CPU. For graphical and parallel computation, an NVIDIA RTX 2080 Ti with 11 GB of VRAM was used. 64GB of RAM and OS Ubuntu 18.04.5 LTS 64 bit also powered the workstation.

### 3.2. Pedestrian Detection Performance Using Different Image Sources

This subsection will explain the comparison between the single image source-based detection and the multispectral image-based detection by using the original YOLO structure. For other object detection tasks, this evaluation metric can depend on the purpose. In this research, the balanced precision-recall value is used to assess the detection accuracy performance. Thus, this study assumes accuracy as the balanced precision-recall value. It means the values where both precision and recall hit the same values. 

**Figure 5:**
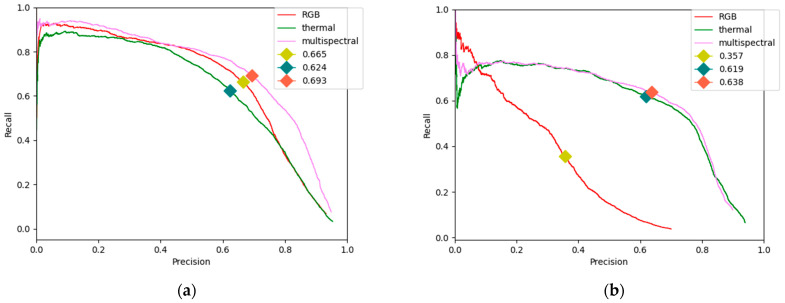
Detection accuracy comparison based on the different image sources between (**a**) daytime and (**b**) nighttime.

The experimental results can be seen in this Figure 5. The red line indicates the precision-recall curve of RGB image-based pedestrian detection. The green line indicates the precision-recall curve of thermal image-based pedestrian detection. Finally, the violet line indicates the precision-recall curve of multispectral image-based pedestrian detection. Additionally, diamonds indicate the balanced precision-recall values, respectively. When the multispectral-based image is used, the performance increases by about 2% compared to the RGB image-based detection performance and about 7% compared to the thermal image-based detection performance during daytime. On the other hand, pedestrian detection at nighttime shows poor performance for the RGB image-based method, with only 35.7% accuracy. Thermal image-based detection at nighttime achieves 61.9% accuracy. Similar to the daytime performance, the multispectral- image based detection hits the best performance at 63.8% accuracy.

Indeed, there have been various common object detection algorithms which can run well for pedestrian detection tasks during the daytime. However, they do not perform well when facing a nighttime environment. Therefore, a thermal image can be a supplementary method to add more information, so the classifier can detect objects more accurately. Now, RGB information is still required at nighttime performance, because at some certain conditions, a person can look like a similar object. If only a thermal picture is fetched, any color details will be invaluable which can lead to misdetection. Nevertheless, if an RGB image is also fetched to the classifier, an additional detail such as the shirt color can give more information to the classifier. 

Moreover, as mentioned before, thermal information is still necessary for daytime environments. More people can be distinguished correctly when utilizing multispectral images. Although it is enough to detect one person as the car can notice there is a person nearby, but in the traffic system domain which involves human’s lives the highest accuracy possible should be achieved.

### 3.3. The YOLO Optimization Experimental Results towards the Detection Accuracy

An optimized model called YOLO-4L was built. The original YOLO method is named YOLO-3L. The same evaluation metric is still used to assess the detection performance. A point to remember, is that all experiments used multispectral images only.

Table 2 compares the detection performance between YOLO-4L and YOLO-3L. In both situations, YOLO-4L shows higher accuracy. In the daytime scenario, the detection performance of YOLO-4L is increased by 2.8%, and the nighttime detection performance is increased by 0.4%. This result indicates that adding more feature output at the earlier residual blocks can improve the pedestrian detection accuracy. People in the images can be recognized by the YOLO-4L model better than with the YOLO-3L model.

After improving the model accuracy, a unified model is proposed. Beforehand, a base model needs to be chosen by doing a preliminary experiment. This preliminary experiment compares the performance between the RGB-based and the thermal-based unified models. The result shows that the thermal-based unified model is a fair model for the base model, because the RGB-based model performance, especially during the nighttime, is too poor. Finally, unified model experiments were conducted and the results shown in Table 3. To ensure algorithm stability, each setup was trained and validated five times from scratch. The final evaluation is determined by averaging validation results of five training instances.

Referring to Table 3, the YOLO-4L multispectral-based model has the best performance among three different models. Compared to the base model, the performance is improved by 6.9%. This is because when the daytime and the nighttime datasets are combined, the model also acquires a new ability to adapt to more diverse cases. Moreover, it is also proved that the optimized model is also stable as in each training phase the accuracy performance has always the best performance among three comparison models. Although the multispectral-based model of YOLO-4L is the best model of the three, there are different interpretations between the thermal model and the multispectral model.

The unified thermal-based model has better detection performance than the independent thermal-based model during both daytime and nighttime. The performance of the thermal-based model during daytime only hits 62.4% and 61.9% during the nighttime. On the other hand, the unified model raises the detection performance to 64.5% on average. This is because when the daytime and nighttime datasets are combined, the classifier does not treat daytime and nighttime as different cases. Thermal images for daytime and nighttime are similar regardless of the lighting conditions, although they are not identical. Consequently, when a nighttime dataset is added into the daytime dataset, the model gets more cases to learn from. Therefore, the unified model result increases around 3% from the independent model’s result of daytime and nighttime.

In contrast, when evaluating the performance of the multispectral-based model, the unified models hit the performance within the result shown from the independent daytime and nighttime of the multispectral-based model. The independent daytime model of the YOLO-3L obtains a detection performance of 69.3%, and the independent nighttime model of the YOLO-3L obtains a detection performance of 63.8%, and the unified model of the YOLO-3L hits a detection performance at the average of 70.13%. Similarly, for the YOLO-4L, the unified model shows an average 71.4% detection performance while the independent daytime model of the YOLO-4L reaches 72.1% of detection performance and the independent nighttime model of the YOLO-4L reaches 64.2% of detection performance. It is not the same event as the thermal-based model result. The multispectral images consist of both RGB image channels and thermal image channels. From the preliminary testing, it has been discovered that RGB-based model performance at nighttime is the worst of all configuration testing results. Although there is supplementary information from the thermal images in multispectral images based, poor detection performance in the RGB-based model performance at nighttime still contributes to the multispectral images generally. 

**Figure 6:**
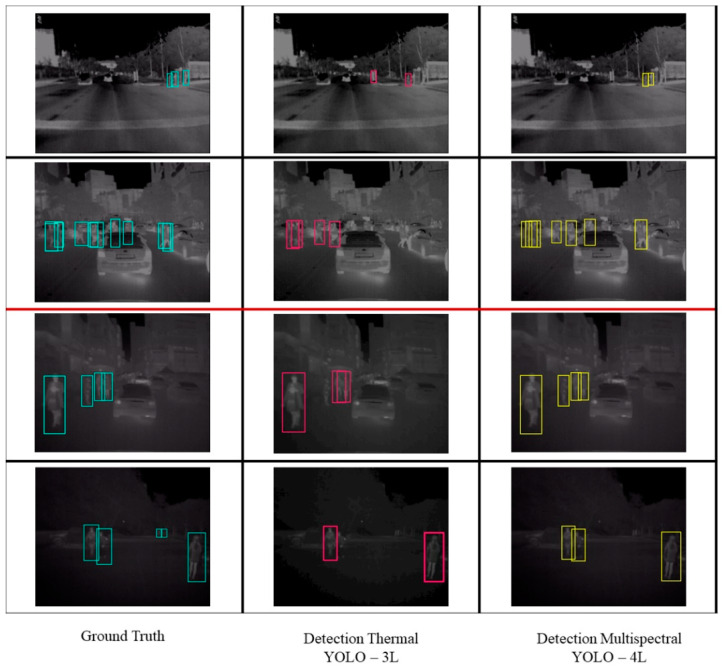
Ground truth and detection examples using the thermal-based of YOLO-3L model and the multispectral-based of YOLO-4L model.

Several detection examples are presented in Figure 6. The first two rows of pictures are daytime scenario examples, and the remaining two pictures are the nighttime scenarios. The first column represents the ground truth labels in each picture. The second column represents the detection result by using the unified thermal-based of YOLO-3L model. The third column represents the detection result from the unified multispectral-based of YOLO-4L model. As seen in Figure 6, some pedestrians have been already detected in the unified thermal-based model. Nevertheless, when using the unified multispectral-based of YOLO-4L model, the number of correct detections is increased. Furthermore, pictures in the first row show that the unified thermal-based model of YOLO-3L misclassified a tree as a person. However, the unified multispectral-based model of YOLO-4L can classify the persons correctly.

### 3.4. The YOLO Optimization Experimental Results towards the Processing Time

**Figure 7:**
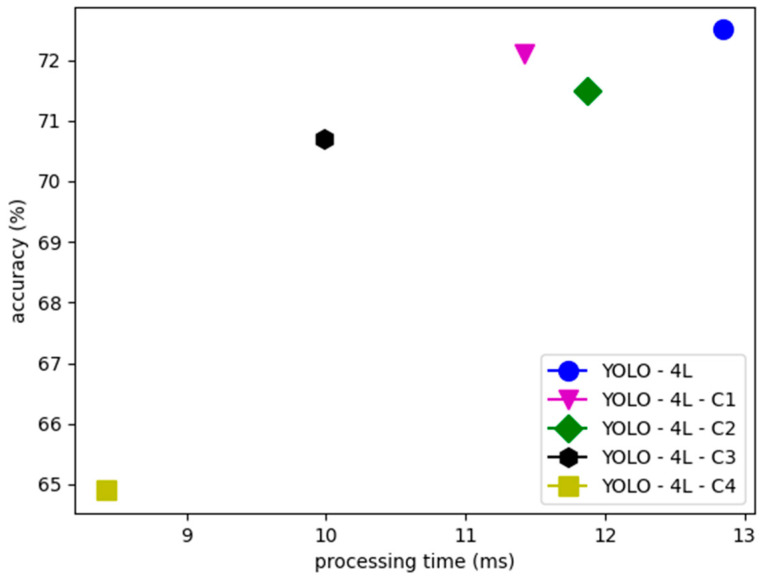
Processing time and accuracy comparison of the YOLO compressed models.

The optimized models of YOLO for improving the processing time are depicted in Figure 7. A blue circle indicates the unified model of YOLO-4L from the previous optimization, the magenta triangle marks the first modified type (C1), the green diamond marks the second modified type (C2), the black hexagon marks the third modified type (C3), and the fourth modified type (C4) is marked by the yellow square. Accuracy in this figure still utilizes the same metric as used in the previous comparison that is called balanced precision and recall.

From the results, indeed the original YOLO-4L achieves the highest accuracy among all compressed models. At first, the C1 model can process the detection faster than the original model, as well as the C2 model by maintaining the accuracy above 71%. However, as the residual blocks and the feature output layers are also reduced, the processing time and the accuracy also decrease. The worst performance is displayed by the C4 model where the accuracy is impaired by 7.6%, although the processing time achieved is less than 9 ms. From these four modifications, the C3 model is proposed as the balanced compressed model of YOLO-4L. The reason is because the processing time is faster by 3 ms and the accuracy can be maintained above 70%.

## 4. Conclusions

An evaluation of pedestrian detection tasks by using deep neural network and multispectral images has been conducted. The first contribution shows that the multispectral image can be the best solution to solve pedestrian detection problems, especially in low-light conditions. A comparison of all different image sources by using the balanced precision-recall metric was shown in this paper, which has never been done before in previous research to the best knowledge of the researchers. 

The second contribution of this research is proposing an optimization of YOLO v3 for improving the detection accuracy. Additional feature outputs were created in the earlier YOLO v3 architecture, so the model can recognize more small pedestrians. From the two proposed models, the four feature output layers or YOLO-4L are the best model, because both daytime and nighttime performances are increased compared to the original YOLO v3 model. 

Finally, another optimization was also proposed for improving the processing time. The compressed model is very important, especially when being implemented on a small-scale device, such as a GPU development board or in a real vehicle. Based on several proposed models, the optimal model has 22.76% improvement in the processing time but still maintains an accuracy at 70.7%. Hence, pedestrian detection task does not always require a huge neural network to achieve good results with slow processing time.

In the future, an augmented tracking algorithm can be used to improve detection accuracy. This algorithm can be combined with the YOLO algorithm. 

The proposed pedestrian detection system can be used in the human-driving cars as a function of ADAS. When pedestrians are detected in the driving direction of the vehicle, the vehicle driver will be informed with an appropriate warning mechanism, such as audio. In this process, the pedestrian detection result, and the vehicle dynamic information (e.g., speed and driving direction) should be considered together to assess the risk level of pedestrians, then only the dangerous pedestrians will trigger the warning mechanism. The proposed pedestrian detection system can also be used in autonomous vehicles. Without warning the driver, the pedestrian detection result will directly be used for controlling the vehicle by the autonomous driving system.

## Figures and Tables

**Table 1:** YOLO optimization architecture comparison models.

YOLO-4L			YOLO-4L-C1			YOLO-4L-C2			YOLO-4L-C3			YOLO-4L-C4		
Layer	Filters	Size	Layer	Filters	Size	Layer	Filters	Size	Layer	Filters	Size	Layer	Filters	Size
Conv	32	3 × 3	Conv	32	3 × 3	Conv	32	3 × 3	Conv	32	3 × 3	Conv	32	3 × 3
Conv	64	3 × 3	Conv	64	3 × 3	Conv	64	3 × 3	Conv	64	3 × 3	Conv	64	3 × 3
Conv	32	1 × 1	Conv	32	1 × 1	Conv	32	1 × 1	Conv	32	1 × 1	Conv	32	1 × 1
Conv	64	3 × 3	Conv	64	3 × 3	Conv	64	3 × 3	Conv	64	3 × 3	Conv	64	3 × 3
1 × Residual Block			1 × Residual Block			1 × Residual Block			1 × Residual Block			1 × Residual Block		
Conv	128	3 × 3	Conv	128	3 × 3	Conv	128	3 × 3	Conv	128	3 × 3	Conv	128	3 × 3
Conv	64	1 × 1	Conv	64	1 × 1	Conv	64	1 × 1	Conv	64	1 × 1	Conv	64	1 × 1
Conv	128	3 × 3	Conv	128	3 × 3	Conv	128	3 × 3	Conv	128	3 × 3	Conv	128	3 × 3
2 × Residual Block			2 × Residual Block			2 × Residual Block			2 × Residual Block			2 × Residual Block		
5 × Feature Output			1 × Feature Output			5 × Feature Output			1 × Feature Output			1 × Feature Output		
Conv	256	3 × 3	Conv	256	3 × 3	Conv	256	3 × 3	Conv	256	3 × 3	Conv	256	3 × 3
Conv	128	1 × 1	Conv	128	1 × 1	Conv	128	1 × 1	Conv	128	1 × 1	Conv	128	1 × 1
Conv	256	3 × 3	Conv	256	3 × 3	Conv	256	3 × 3	Conv	256	3 × 3	Conv	256	3 × 3
8 × Residual Block			8 × Residual Block			4 × Residual Block			4 × Residual Block			4 × Feature Output		
5 × Feature Output			1 × Feature Output			5 × Feature Output			1 × Feature Output			1 × Feature Output		
Conv	512	3 × 3	Conv	512	3 × 3	Conv	512	3 × 3	Conv	512	3 × 3	Conv	512	3 × 3
Conv	256	1 × 1	Conv	256	1 × 1	Conv	256	1 × 1	Conv	256	1 × 1	Conv	256	1 × 1
Conv	512	3 × 3	Conv	512	3 × 3	Conv	512	3 × 3	Conv	512	3 × 3	Conv	512	3 × 3
8 × Residual Block			8 × Residual Block			4 × Residual Block			4 × Residual Block			2 × Residual Block		
5 × Feature Output			1 × Feature Output			5 × Feature Output			1 × Feature Output			1 × Feature Output		
Conv	1024	3 × 3	Conv	1024	3 × 3	Conv	1024	3 × 3	Conv	1024	3 × 3	Conv	1024	3 × 3
Conv	512	1 × 1	Conv	512	1 × 1	Conv	512	1 × 1	Conv	512	1 × 1	Conv	512	1 × 1
Conv	1024	3 × 3	Conv	1024	3 × 3	Conv	1024	3 × 3	Conv	1024	3 × 3	Conv	1024	3 × 3
4 × Residual Block			4 × Residual Block			4 × Residual Block			4 × Residual Block			2 × Residual Block		
6 × Feature Output + SPP			1 × Feature Output + SPP			6 × Feature Output + SPP			1 × Feature Output + SPP			1 × Feature Output + SPP		

**Table 2:** Detection performance between the optimized and the original YOLO models.

Model	Daytime	Nighttime
YOLO-3L	69.3%	63.8%
YOLO-4L	72.1%	64.2%

**Table 3:** Unified model comparison among thermal-based (YOLO–3L) and multispectral-based (YOLO–3L and YOLO–4L).

Configuration		Training					Average
		1st	2nd	3rd	4th	5th	
YOLO-3L	Thermal	65.4%	63.9%	63.9%	63.8%	64.9%	64.5%
	Multispectral	69.6%	70.7%	69%	71.2%	70.7%	70.13%
YOLO-4L	Multispectral	71.2%	71.7%	71.2%	72.5%	71.8%	71.4%

## Data Availability

Not applicable.
